# Financial perceptions and subjective well-being among older adults

**DOI:** 10.3389/fpsyg.2025.1709795

**Published:** 2026-01-06

**Authors:** Maryam Jafari Bidgoli, Abbey Gregg, Catanya G. Stager, Martha Crowther

**Affiliations:** 1 Department of Community Medicine and Population Health, College of Community Health Sciences, The University of Alabama, Tuscaloosa, AL, United States; 2 Division of General Internal Medicine and Population Science, Heersink School of Medicine, The University of Alabama at Birmingham, Birmingham, AL, United States

**Keywords:** subjective financial well-being, subjective well-being, hopelessness, positive social support, negative social support, older adults, financial psychology

## Abstract

**Objective:**

To examine how subjective financial well-being (SFWB) relates to subjective well-being (SWB) among older adults in the U.S. Beyond material resources, SFWB reflects perceptions of present and future security, stability, and freedom of choice. This study tested whether hopelessness mediates the SFWB–SWB relationship and whether perceived social support, both positive (PSS) and negative (NSS), moderates this pathway.

**Methods:**

Data came from 4,570 respondents (Mean age = 69; 56% female) in the Health and Retirement Study, a nationally representative panel survey of U.S. adults over age 50, sponsored by the National Institute on Aging. SFWB was measured using the Consumer Financial Protection Bureau’s (CFPB) 10-item scale, a multidimensional instrument that captures not only current financial security but also expectations for future financial stability and control. SWB was measured using Diener’s Satisfaction With Life Scale. Hopelessness was assessed with a 4-item index, and perceived social support was measured across four relational domains (spouse, children, family, friends), using 28 items divided into PSS and NSS. Analyses were conducted using PROCESS (v5.0) in SPSS, controlling for sociodemographic factors.

**Results:**

SFWB was positively associated with SWB and negatively associated with hopelessness. Hopelessness significantly mediated the SFWB–SWB link, accounting for 24.4% of the total effect. NSS significantly moderated the SFWB–hopelessness pathway, amplifying the indirect effect of low SFWB on SWB. PSS had a weaker but still significant moderating effect. When both forms of support were modeled simultaneously, only NSS remained a significant moderator.

**Conclusion:**

SFWB significantly contributes to older adults’ life satisfaction, partly by reducing hopelessness. This multidimensional measure is important in aging research because it reflects not just income or assets but also perceived financial security, stability, and the freedom to make choices in the present and future. Negative social support intensifies the psychological toll of financial insecurity, while positive support provides limited buffering. Interventions should address both financial perceptions and social relationships to strengthen resilience and well-being in later life.

## 1 Introduction

Subjective financial well-being (SFWB), an individual’s perception of financial security and control, plays an important role in shaping subjective well-being (SWB) as people age. For older adults, financial concerns often extend beyond income to include autonomy, stability, and future preparedness. Traditional sources of support shift in later life from decreasing income from work and increasing dependence on Social Security and personal savings. As such, SFWB becomes a key psychological resource linked to life satisfaction, emotional resilience, and overall quality of life. For example, for older adults, financial strain results in decreasing subjective health and functional capacity to take care of themselves (Angel et al., 2003).

SWB is a foundational construct in the study of well-being, reflecting how individuals evaluate the overall quality of their lives (Diener et al., 2018). It is typically measured through both cognitive appraisals—such as life satisfaction across domains like work and family—and affective experiences, including positive and negative emotions (Diener, 2000; Pavot, 2018). Researchers have employed a range of validated tools to assess SWB (Diener, 1984; Diener et al., 1985; Gadermann et al., 2010; Huebner, 1994; Kitayama and Markus, 2000; Lyubomirsky and Lepper, 1999).

Building on this conceptual foundation, recent scholarship has increasingly examined the role of financial determinants in shaping SWB, particularly across the aging process. Growing attention has been directed toward the role of financial factors such as income, socioeconomic status, and financial satisfaction in shaping SWB across the lifespan (Tan et al., 2020). Despite this progress, many studies still rely heavily on global assessments of SWB, often overlooking how satisfaction within specific life domains contributes to overall well-being. Recent research has emphasized the need for a domain-specific approach, highlighting that different life areas such as health, social relationships, and financial stability may show distinct patterns and impacts across the life course (Bardo, 2017).

Financial well-being (FWB) has been recognized as a significant determinant of life satisfaction, garnering increasing attention in both economic research and public policy domains (Arber et al., 2014; Nanda and Banerjee, 2021; Ngamaba et al., 2020). While FWB and life satisfaction are frequently conceptualized as interrelated and mutually reinforcing, the exact nature of their association remains a subject of ongoing debate (Diener et al., 2018; Ngamaba et al., 2020). Among FWB research, a lack of consensus persists regarding a comprehensive theoretical framework. Some studies distinguish between objective FWB (e.g., income, assets) and subjective FWB (e.g., perceived financial control and security) (Arber et al., 2014), whereas others propose broader models incorporating psychological, behavioral, and relational components (Gerrans et al., 2013; Joo and Grable, 2004; Sorgente and Lanz, 2017; Zemtsov and Osipova, 2016). Importantly, subjective perceptions of FWB may yield outcomes that differ meaningfully from those based solely on objective financial status (Erner et al., 2016). In 2015, the U.S. Consumer Financial Protection Bureau (CFPB) (Consumer Financial Protection Bureau, 2015) initiated research aimed at defining and explaining individuals’ subjective perceptions of FWB. From CFPB’s report, FWB is defined as “*a state of being wherein you have control over day-to-day, month-to-month finances; have the capacity to absorb financial shock; are on track to meet your financial goals; and have the financial freedom to make the choices that allow you to enjoy life*” (Consumer Financial Protection Bureau, 2015, p. 5). For older adults, feeling in control of their finances allows them to pursue leisure activities; this freedom to pursue enjoyable activities can strengthen social networks, emotional well-being, and is associated with higher life satisfaction (Finke et al., 2017; Parra-Rizo et al., 2022).

While income and wealth have been widely studied as predictors of SWB (Deaton, 2008; Diener et al., 1993; Jebb et al., 2018; Lin et al., 2024; Stevenson and Wolfers, 2013) fewer studies have explored the unique role of financial perceptions, especially in later life (Iannello et al., 2021; Yeo and Lee, 2019). Although FWB has been broadly linked to SWB (Arber et al., 2014), much less is known about how financial perceptions specifically interact with psychological constructs to shape well-being among older adults. Understanding SFWB is particularly important for older adults because perceptions of financial control and security may influence life satisfaction more than objective measures. This gap points to an important and underexplored area of inquiry: the interaction between financial perceptions, psychological resources, and life satisfaction in later life.

## 2 Literature review

### 2.1 Financial well-being and aging

As individuals age, FWB increasingly functions as a core component of psychological adjustment. Older adults face unique financial stressors, including fixed incomes, rising healthcare costs, and uncertainty regarding long-term care, which often compound vulnerabilities in other life domains (DeVaney, 2008). These challenges are not solely economic but psychological, as perceptions of financial insecurity have been linked to diminished emotional resilience and reduced life satisfaction in later life (Arber et al., 2014; Yeo and Lee, 2019). With demographic trends indicating sustained growth in the aging population, there is a critical need to understand the psychosocial mechanisms that link financial perceptions with broader outcomes such as life satisfaction.

Financial anxiety (i.e., persistent worry about current or future financial stability) has emerged as a significant mental health concern among older adults. Unlike material deprivation, financial anxiety stems from subjective perceptions of vulnerability and a lack of control over one’s financial future. Older adults who feel uncertain about their ability to meet future expenses often report elevated stress, depressive symptoms, and poorer sleep quality, even when their objective financial situation is relatively stable (Guan et al., 2022; Hall et al., 2008; Ryu and Fan, 2023). This anxiety is compounded by age-related declines in financial literacy, health, and cognitive functioning, which can reduce individuals’ confidence in managing money and navigating financial decisions (Boyle et al., 2025; Gamble et al., 2015). As a result, financial anxiety not only undermines psychological well-being but also erodes older adults’ sense of agency, leaving them less equipped to cope with the challenges of aging.

Given these dynamics, understanding how perceived financial control and security influence emotional states—and ultimately overall life satisfaction—offers valuable insight into the psychological processes that shape well-being in later life. This perspective underscores the importance of examining financial perceptions not merely as economic indicators, but as core determinants of psychological health among older adulthood. In this study, FWB is used as a broad term, whereas SFWB refers specifically to individuals’ perceptions of their financial well-being, particularly their sense of financial security and control.

### 2.2 The mediation role of hopelessness

Hopelessness is commonly defined as a cognitive state involving negative expectations about the future and a perceived inability to achieve personally meaningful goals (Beck et al., 1974; Abramson et al., 1989). It may also involve the absence of clear strategies or pathways for goal attainment, as suggested by psychological research originally focused on hope (Mitchell et al., 2020). While hope has been consistently identified as a key psychological factor associated with SWB, with higher levels of hope linked to increased life satisfaction and overall happiness (Karatas et al., 2021; McConnell and Stull, 2017; Oliver et al., 2017), its absence—hopelessness—has been linked to diminished well-being, often manifesting as despair, lack of purpose, and disengagement.

Within the financial domain, hopelessness may arise when individuals perceive their financial situation as unstable or inadequate to meet future needs. Prior research suggests that financial satisfaction can foster hope by serving as a psychological resource that promotes a more positive future oriented outlook. Similarly, financial security enables individuals to form optimistic expectations about their future and may act as a buffer against hopelessness, thereby supporting overall well-being. Furthermore, improvements in FWB can enhance life satisfaction by reducing present financial strain and reinforcing a sense of future stability and control (Loewenstein, 1987; Pleeging et al., 2020).

These findings support the conceptualization of hopelessness as a potential mechanism linking financial perceptions to life satisfaction, particularly in later life when concerns about financial stability and future security often become more salient. While current evidence on financial perceptions is limited, it is plausible to suggest that SFWB influences SWB through hopelessness, positioning it as a candidate mediator in this relationship.

### 2.3 The moderating role of social support

A strong network of friends, family, and peers can provide emotional reassurance, practical support, and a sense of belonging, which in turn can help individuals navigate difficult times and maintain a more hopeful outlook on the future (Goldzweig et al., 2016; Hassan-Ohayon et al., 2014). The absence of close social ties has been linked to adverse physical and psychological health outcomes (Lincoln, 2000). Social support is commonly understood in two forms: *received support* (tangible aid from others) and *perceived support* (the belief that support is available when needed). Perceived support reflects the perception that support would be accessible whenever needed (Uchino et al., 2018). Compared to received support, perceived support is a stronger and more consistent predictor of psychological resilience and hope. It has been identified as a key indicator of life satisfaction across various populations, including older adults (Barrera, 1986; Şahin et al., 2019; Wu et al., 2022), and is closely associated with overall well-being (Yeo and Lee, 2019). Individuals who perceive strong support often feel more confident in accessing needed resources, which can buffer against hopelessness and reduce emotional distress (Mo et al., 2014; Zuo et al., 2021).

While most research has traditionally focused on the benefits of positive social support, few studies have examined its negative dimensions (Lincoln, 2000). However, in a smaller body of research that examined both sides, there is a mix of findings regarding their effects on well-being. Some studies suggest that positive interactions have a stronger impact, while others propose that negative social experiences could have a more powerful effect on well-being than positive ones (Abbey et al., 1985; Davis and Rhodes, 1994). Nonetheless, empirical data indicates that negative interactions might carry a greater potential for harm compared to the beneficial impact of social support (Lincoln, 2000). Moreover, social support mitigates feelings of hopelessness among individuals facing challenging circumstances (Xiang et al., 2020). Taken together, the evidence suggests that while positive support enhances resilience, negative interactions may exert disproportionately stronger effects on well-being.

## 3 Current study

SWB comprises three distinct components: (1) affect (e.g., positive and negative emotions), (2) domain-specific satisfaction (e.g., satisfaction with family, friends, health, hobbies, and place of residence), and (3) global evaluations (e.g., overall life satisfaction and happiness) (Bardo, 2017). The present study focuses on the third component, a well-established indicator of self-evaluated life quality. This study makes a unique contribution to the literature by examining how the subjective aspect of FWB shapes global life satisfaction in adults aged 50 and older, and by exploring how psychological and social resources—specifically hopelessness and perceived social support—mediate and moderate this relationship.

This study had three aims: (1) to examine the direct relationship between SFWB and SWB, (2) to test whether hopelessness mediates this relationship, and (3) to assess whether perceived social support (PSS, NSS) moderates the indirect effect. To address these aims, a series of moderated mediation models are employed to assess both the direct and indirect effects of SFWB on SWB. These analyses aim to clarify the psychological and social mechanisms linking financial perceptions to well-being in later life. Based on this framework, the following hypotheses are proposed:

H1: Higher SFWB will be associated with higher SWB (direct effect).

H2: Hopelessness will mediate the SFWB-SWB relationship.

H3a: Higher NSS will amplify the indirect effect of SFWB on SWB via hopelessness.

H3b: Lower PSS will amplify the indirect effect of SFWB on SWB via hopelessness.

Although positive and negative support are typically studied separately, research suggests their effects may interact (Rook, 2015; Lincoln, 2000). Accordingly, we extend our hypotheses to consider both independent and interactive moderation.

H3c: When modeled independently, NSS and PSS will each moderate the SFWB–hopelessness pathway.

H3d: When modeled interactively, the moderating role of NSS will depend on PSS.

## 4 Method

### 4.1 Transparency and openness

The study design and analytic plan were not preregistered. The data used were from the open-access, publicly available Health and Retirement Study (HRS). The HRS began in 1992 and biennially surveys a representative sample of approximately 20,000 people in America aged 50+. The HRS is sponsored by the National Institute on Aging (grant numbers NIA U01AG009740 and NIA R01AG073289) and is conducted by the University of Michigan. All data and measures are publicly available on the HRS website, ensuring full reproducibility of results: https://hrsdata.isr.umich.edu/data-products/public-survey-data.

The method section describes how sample size, any data exclusions, any manipulations, and all measures were determined. Stata/MP 16.1 was used for data preparation and computation of the SFWB composite scores. Details on predicting SFWB scale scores, including guidelines for using the Stata PFWB module developed by Nichols (2017), are provided in the Supplementary material. *SPSS Statistics* (v29.0) with the PROCESS macro v5.0 was used for analytic models. Instructions for installing the PROCESS macro are also included in the Supplementary material. Given that HRS data are publicly available and deidentified, our study did not require review from the University of Alabama, Institutional Review Board.

### 4.2 Participants and procedure

The sample included 4,570 respondents aged 50+, drawn from the 2020 HRS Psychosocial and Lifestyle Questionnaire. The HRS is a nationally representative, panel study of older adults in the United States (Juster and Suzman, 1995). This study specifically used the 2020 HRS Psychosocial and Lifestyle Questionnaire (Leave Behind HRS core data file, March 2020–May 2021) (Health and Retirement Study, 2020) to construct scales for SFWB, hopelessness index, PSS and NSS, and used the RAND HRS Longitudinal File 2022 (V1) for SWB, and covariates (Health and Retirement Study, 2025; RAND Center for the Study of Aging, 2025). The HRS Psychological and Lifestyle Questionnaire is in the form of a self-administered questionnaire which was provided to respondents after they had finished an in-person Core Interview. This psychosocial information is obtained during each biennial wave from a randomly selected 50% of the core panel participants who engage in the comprehensive face-to-face interview.

Of the total sample (*N* = 4,570), a subset of 4,234 had complete data across all analytic variables. A detailed description of the study participants is provided in Supplementary Figure S1. The mean age of participants was 69 years (*SD* = 10.1). 60% identified as female, and 26% were aged 50 to 61 years. Participants self-identified as White/Caucasian (71%), Black/African American (19%), or other racial backgrounds (10%) (Table 1). A detailed description of the covariates is provided in Supplementary Table S1. All analyses were conducted using the PROCESS v5.0 macro in *SPSS*, which applies listwise deletion by default. Prior to model estimation, the dataset was examined for missingness, which was minimal (ranging from 0.02 to 2.4%). The extent of missing data across the study variables is reported in Supplementary Table S2.

**Table 1:** Descriptive statistics and correlation coefficients.

Variable	*n*^a^	M (%)^b^	SD	Minimum	Maximum	Pearson correlation				
						1	2	3	4	5
1. SWB	4,506	5.03	1.47	1	7	—	0.40***	−0.36***	0.31***	−0.27***
2. SFWB	4,459	63.01	13.78	14	95		—	−0.38***	0.18***	−0.30***
3. Hopelessness scale	4,507	2.21	1.22	1	6			—	−0.28***	0.26***
4. PSS	4,469	3.18	0.53	1.11	4				—	−0.32***
5. NSS	4,479	1.59	0.46	1	3.88					—
PSS (spouse/partner)	2,922	3.48	0.66	1	4					
PSS (children)	3,947	3.29	0.74	1	4					
PSS (family)	4,271	2.96	0.87	1	4					
PSS (friends)	4,034	3.12	0.73	1	4					
NSS (spouse/partner)	2,917	1.91	0.67	1	4					
NSS (children)	4,016	1.64	0.62	1	4					
NSS (family)	4,273	1.56	0.63	1	4					
NSS (friends)	4,026	1.37	0.48	1	4					
Age	4,569	69.28	10.01	50	99					
Age 50–61	1,193	26.11								
Age ≥ 62	3,376	73.87								
Gender										
Female	2,752	60.22								
Male	1,818	39.78								
Race										
White/Caucasian	3,244	70.98								
Black/African American	875	19.15								
Other^c^	433	9.47								
Ethnicity										
Hispanic	639	13.98								
Not Hispanic	3,927	85.93								
Marital status										
Married	2,832	61.97								
Not married	1,723	37.70								
Education										
High school or less/GED	2,013	44.05								
Some college	1,238	27.09								
College and above	1,319	28.86								
Household poverty threshold										
Below	484	10.59								
Above	4,058	88.80								

SWB, subjective well-being; SFWB, subjective financial well-being; PSS, positive social support; NSS, negative social support. ***p < 0.001.

a Of the total sample (*N* = 4,570), a subset of 4,234 had complete data across all variables.

b M (%) = Mean for continuous variables; percentage for categorical variables.

c Other includes American Indian, Alaskan Native, Asian, Native Hawaiian, and Pacific Islander.

### 4.3 Measures

#### 4.3.1 Subjective financial well-being

##### 4.3.1.1 Instrument and dimensions

SFWB was assessed using the framework developed by the CFPB (Consumer Financial Protection Bureau, 2017), which defines FWB as a state in which individuals can meet current and ongoing financial obligations, feel secure about their financial future, and have the freedom to make choices that enhance their quality of life. The scale was designed to assess multiple dimensions of FWB beyond income or wealth alone. The CFPB model outlines four key dimensions: (1) Control over day-to-day finances (2) Capacity to absorb financial shocks (3) Progress toward financial goals, and (4) Financial freedom to make life choices. This conceptualization captures both cognitive and emotional aspects of financial well-being, aligning closely with broader measures of life satisfaction (Riitsalu et al., 2024).

##### 4.3.1.2 Response scale

The CFPB financial well-being scale contains 10 items. Responses were recorded on two 5-point Likert scales: six items ranged from “Completely” to “Not at all,” and four items ranged from “Always” to “Never.” The items assess various financial experiences, including the ability to handle unexpected expenses, confidence in long-term security, satisfaction with money management, current financial stress, difficulty meeting expenses, financial aspirations, surplus resources, falling behind, and perceived influence of finances on one’s life (see Table 2).

**Table 2:** CFPB subjective financial well-being scale.

Items	Responses options
1. I could handle a major unexpected expense.	4 = Completely 3 = Very well 2 = Somewhat 1 = Very little 0 = Not at all
2. I am securing my financial future.	
3. I can enjoy life because of the way I’m managing my money.	
4. Because of my money situation, I feel like I will never have the things I want in life.	
5. I am just getting by financially.	
6. I am concerned that the money I have or will save won’t last.	
7. Giving a gift for a wedding, birthday or other occasion would put a strain on my finances for the month.	0 = Always 1 = Often 2 = Sometimes 3 = Rarely 4 = Never
8. I have money left over at the end of the month.	
9. I am behind with my finances.	
10. My finances control my life.	

The 10-item scale developed by the CFPB to assess individuals’ perceived financial well-being.

##### 4.3.1.3 Scoring and reliability

The Stata PFWB package developed by Nichols (2017) was used to calculate SFWB scale scores based on the Consumer Financial Protection Bureau (2017) survey instrument. Prior to scoring, item responses were recorded on a 0–4 scale and reverse coded if needed, with higher values consistently representing more favorable FWB. Following the CFPB’s scoring guidelines (see Supplementary material), FWB scores were derived using item response theory, which provides a more precise and psychometrically robust estimation than simple summation or averaging. Scores range from 0 (worse) to 100 (best), and the scoring algorithm also adjusts for respondent age by differentiating between those under age 62 and those 62 or older, accounting for life-stage financial contexts (Consumer Financial Protection Bureau, 2017, 2018). While the PFWB approach can estimate scores for respondents with incomplete data using IRT-based priors, we opted to set the SFWB score to missing for individuals with four or more missing items to ensure consistency and reliability across all measures used in the study (Cronbach α = 0.89).

#### 4.3.2 Subjective well-being

SWB reflects individuals’ overall cognitive evaluation of their life satisfaction. It was measured using the 5-item Satisfaction with Life Scale developed by Diener et al. (1985) and later validated by Pavot and Diener (1993) (Cronbach α = 0.87). Participants rated their agreement with each item (e.g., “In most ways my life is close to my ideal”) on a 7-point Likert scale ranging from “1 = *Strongly disagree*” to “7 = *Strongly agree*” (see Appendix A). An index was created by averaging the scores across all 5 items. The final score was set to missing if there were three or more items with missing values. This index was created by RAND HRS (2025), as described in the Psychosocial and Lifestyle Questionnaire (2006–2022) User Guide (Smith et al., 2023).

#### 4.3.3 Hopelessness

Hopelessness reflects negative expectations about future and a sense of futility regarding one’s goals and desire. It was measured using four items. Two items came from Everson et al. (1997), and assessed individuals’ sense that achieving future goals is impossible and their belief in a hopeless future. The other two items came from Beck et al. (1974), and captured expectations about realizing personal wishes and the perceived futility of striving toward them (Cronbach α = 0.85). Responses to a minimum of two of the four items were needed to construct the scale, which was averaged such that higher scores represented greater feelings of hopelessness (see Appendix B).

#### 4.3.4 Perceived social support

Perceived social support reflects the quality of social relationships and the extent of perceived support that individuals receive from their spouses, children, family, and friends. It was measured as both PSS and NSS using a total of 28 items (Smith et al., 2023). Four sets of 7 items examined the perceived support that respondents received from their spouses, children, family, and friends. For each relationship category, there are 3 positively worded items and 4 negatively worded items. Table 3 reports Cronbach’s α for each relationship category. All items were reverse coded so higher scores reflect greater levels of either PSS or NSS. Then, we created an index of PSS and an index of NSS for each relationship category by averaging the scores within each dimension (i.e., eight scales total). We set the final score to missing if there was more than one item with missing values for the PSS scale, or more than two items with missing values for the NSS scale. Overall PSS and NSS scales were created by aggregating across the relevant relationship categories and were set to missing if more than two relationship category scores were missing (see Appendix C).

**Table 3:** Measure of scale reliability coefficient.

Scale	No. items	Cronbach’s alpha
SFWB	10	0.89
SWB	7	0.88
Hopelessness scale	4	0.85
PSS	12	0.82
PSS, spouse/partner	3	0.81
PSS, children	3	0.84
PSS, family	3	0.86
PSS, friends	3	0.84
NSS	16	0.86
NSS, spouse/partner	4	0.78
NSS, children	4	0.77
NSS, family	4	0.79
NSS, friends	4	0.77

Cronbach’s alpha values indicate internal consistency for each scale used in the analysis. SWB, subjective well-being; SFWB, subjective financial well-being; PSS, positive social support; NSS, negative social support.

## 5 Analytic design

In statistical and social science research, *mediation* explains the mechanism through which an independent variable (SFWB) influences a dependent variable (SWB) via a third variable (hopelessness). *Moderated mediation* occurs when the strength of this indirect effect varies depending on the level of a fourth variable—in this case, perceived social support. Thus, the indirect pathway from SFWB to SWB through hopelessness is conditional on levels of perceived social support. This study further distinguishes between *partial moderated mediation*, where one moderator (e.g., NSS) influences the indirect effect while the second moderator (e.g., PSS) is held constant, and *moderated-moderated mediation*, where the moderation effect itself is conditional on the level of a second moderator (e.g., PSS) (Hayes, 2013; Hayes, 2015; Hayes, 2017; Hayes and Rockwood, 2019).

### 5.1 Statistical models

Equations 1 and 2 represent the most comprehensive form of the moderated mediation framework. These equations include all relevant interaction terms to assess how the indirect effect of SFWB on SWB, via hopelessness (*M*) is simultaneously moderated by both perceived NSS and PSS, as well as their interaction (*WZ*). This first-stage model—where moderation occurs between SFWB and hopelessness—accounts for all main effects and interactions necessary to estimate conditional indirect effects across varying levels of the moderators.


$$\begin{array}{l} M=i_{M}+a_{1}X+a_{2}W+a_{3}Z+a_{4}\mathrm{XW}+a_{5}\mathrm{XZ}+a_{6}\mathrm{WZ}+\\ a_{7}\mathrm{XWZ}+\sum_{j=1}^{7}a_{j+7}U_{j}+e_{M} \end{array}$$ (1)

$$Y=i_{Y}+c\prime X+b_{1}M+\sum_{j=1}^{7}b_{j+1}U_{j}+e_{Y}$$ (2)

Where, *X* is SFWB, *Y* is SWB, *M* is hopelessness. *W* and *Z* represent NSS and PSS, respectively. *U_j_* represents the control variables (e.g., age, gender, race, etc.). *e_M_* and *e_Y_* are error terms. All other models estimated in this study are nested within this framework.

**Figure 1:**
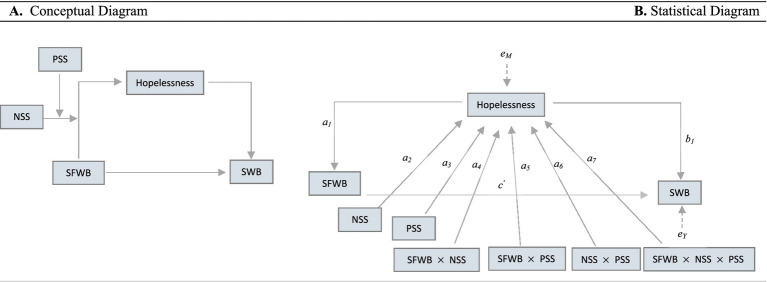
A first stage moderated-moderated mediation model of associations between SFWB and SWB. This figure illustrates Model 4. **(A)** Displays hypothesized associations, with hopelessness serving as the mediator between SFWB and SWB, and NSS and PSS serving as the moderators. **(B)** Presents the coefficient estimates for associations among SFWB, SWB, hopelessness, NSS and PSS. For simplicity, other covariates included in the model are not shown. *e_M_* and *e_Y_* are error terms. Direct effect, c’; Conditional indirect effect of SFWB on SWB through hopelessness, [*a_1_* + *a_4_*(NSS) + *a_5_*(PSS) + *a_7_*(NSS x PSS)]*b_1._*. SFWB, subjective financial well-being; SWB, subjective well-being; NSS, negative social support; PSS, positive social support.

Detailed figures and corresponding equations for Models 1–3 are available in the Supplementary material. These Models each represent simpler forms of the moderated mediation framework (i.e., mediation only, single moderator, and dual independent moderators). Equations 1 and 2 were used to estimate the coefficients for the mediation and outcome regression models. Figure 1 and Supplementary Figures S2–S4 (see Supplementary material) present the conceptual and statistical diagrams for each model. While not shown in the figures, all models included the following covariates: age (*U_1_*; age 50–61, age ≥ 62), gender (*U_2_*; female, male), race (*U_3_*; White/ Caucasian, African American/ Black, Other), ethnicity (*U_4_*; Hispanic, non-Hispanic) and (*U_5_*; married, unmarried), education (*U_6_*; less than high school/ high school or GED, some college, college degree or higher), and poverty level (*U_7_*; below poverty threshold, above poverty threshold). These covariates were included to control for potential confounding influences.

We tested our hypotheses using Hayes’ PROCESS macro: Model 4 (H1–H2: mediation), Model 7 (H3a–H3b: moderated mediation), Model 9 (H3c: partial moderated mediation), and Model 11 (H3d: moderated-moderated mediation).

#### 5.1.1 Mediation (Model 1)

Model 1 estimates a simple mediation model to test whether hopelessness mediates the association between SFWB and SWB. To specify this model, all moderator terms were set to zero (i.e., *W = Z = XW = XZ = WZ = XWZ = 0*) in Equations 1 and 2 (see Supplementary Equations S1, S2). The indirect effect is calculated as *a_1_b_1_* and the direct effect is denoted as *c’* (see Supplementary Figure S2). We formally tested whether hopelessness significantly mediates the relationship between SFWB and SWB.

#### 5.1.2 Moderated mediation (Model 2)

Model 2 introduces a single moderator on the path from SFWB to hopelessness [i.e., a first-stage moderated mediation; Hayes (2013)], using either NSS or PSS as the moderators (see Supplementary Figure S3). For instance, when NSS is specified as the moderator, the terms involving PSS are omitted by setting *Z = XZ = WZ = XWZ = 0* (see Supplementary Equations S3, S4). This model tests whether the indirect effect varies across levels of a single moderator. The conditional indirect effect calculated as *(a_1_ + a_3_W)b_1_*, where *W* represents either NSS or PSS, and the direct effect is *c’*. Separate analyses were conducted for each moderator. A formal test of the index of moderated mediation (Hayes, 2015) was conducted to determine whether the indirect effect is statistically significant across levels of the moderator.

#### 5.1.3 Partial moderated mediation (Model 3)

Model 3 includes both perceived NSS and PSS as independent moderators of the relationship between SFWB and hopelessness (see Supplementary Figure S4). This model assumes no interaction between the moderators by setting the terms *WZ* and *XWZ* to zero (see Supplementary Equations S5, S6) (Hayes, 2017). The first-stage moderation is calculated as *a_1_ + a_4_W + a_5_Z*, where *a_1_* is the effect of SFWB on hopelessness, and *a_4_* and *a_5_* capture the moderation effects of NSS and PSS, respectively. The indirect effect of SFWB on SWB through hopelessness is then calculated as *a_1_b_1_ + a_4_b_1_W + a_5_b_1_Z*, with *b_1_* representing the effect of hopelessness on SWB. The terms *a_4_b_1_* and *a_5_b_1_* represent the partial moderated mediation effects by NSS and PSS. Formal tests were conducted to examine whether these indirect effects changed depending on the level of one moderator while holding the other constant (Table 4, Model 3). An index was considered statistically significant if its bootstrap confidence interval did not include zero (Hayes, 2015).

**Table 4:** Indices of moderated mediation.

Index	Model 2A		Model 2B		Model 3		Model 4	
	Index	95% CI	Index	95% CI	Index	95% CI	Index	95% CI
Index of moderated mediation								
NSS	0.003	[0.001, 0.005]						
PSS			−0.002	[−0.003, 0.000]				
Indices of partial moderated mediation								
NSS					0.003	[0.001, 0.005]		
PSS					0.000	[−0.002, 0.001]		
Indices of conditional moderated mediation by NSS								
Low PSS							0.002	[0.000, 0.005]
Moderate PSS							0.003	[0.001, 0.005]
High PSS							0.004	[0.002, 0.007]
Index of Moderated-Moderated Mediation							0.002	[−0.001, 0.005]

Indices quantify changes in the indirect effect for a one-unit change in the moderator(s). CIs excluding zero indicate significant moderated (or moderated-moderated) mediation. PSS, positive social support; NSS, negative social support. Bootstrap sample size = 10,000; Bootstrap 95% CI, confidence interval.

#### 5.1.4 Moderated-moderated mediation (Model 4)

Model 4 as shown in Figure 1, includes both perceived NSS and PSS as moderators, along with their interaction term, allowing for conditional moderated mediation where the effect of one moderator (e.g., NSS) depends on the level of the other (e.g., PSS) (Hayes, 2017). The conditional indirect effect of SFWB on SWB through hopelessness is calculated as *a_1_b_1_ + (a_4_b_1_ + a_7_b_1_Z)W + a_5_b_1_Z*, using the coefficient estimated from Equations 1 and 2. Here *b_1_* represents the effect of hopelessness on SWB, and *a_4_b_1_ + a_7_b_1_Z* represents how the indirect effect varies with NSS at different levels of PSS. Hayes (2017) refers to this term as the *index of conditional moderated mediation* by *W.* A statistically significant index—indicated by a bootstrap confidence interval that does not include zero—suggests that the strength of the indirect effect of SFWB on SWB via hopelessness depends on the level of PSS. A formal test of the index of moderated-moderated mediation was conducted to determine whether the moderating effect of NSS on the indirect relationship between SFWB and SWB varies as a function of PSS.

Data preprocessing and cleaning were conducted using Stat a MP 16 (StataCorp, 2019). Regression parameters for four specified models were estimated using the PROCESS macro v5.0 for *SPSS Statistics* (v29.0). The macro includes 92 preprogrammed models, and Models 4, 7, 9, and 11 were used in this study. SFWB and the moderator variables were mean-centered prior to creating interaction terms. All indices were tested using 95% bias-corrected bootstrap confidence intervals based on 10,000 resamples. Additionally, the conditional indirect effects of SFWB on SWB were estimated and plotted at the −1 *SD,* 0 *SD,* +1 *SD* (i.e., low, moderate, and high) of NSS or PSS. We tested whether these conditional indirect effects were statistically different from zero at each level of support.

## 6 Results

As expected, SFWB was negatively associated with hopelessness (*r* = −0.378, *p* < 0.001) and perceived NSS (*r* = −0.304, *p* < 0.01) and positively associated with perceived PSS (*r* = 0.175, *p* < 0.001), and SWB (*r* = 0.399, *p* < 0.001). The hopelessness index was negatively associated with perceived PSS (*r* = −0.277, *p* < 0.001) and positively with perceived NSS (*r* = 0.261, *p* < 0.001). Notably, perceived PSS was positively associated with SWB (*r* = 0.313, *p* < 0.001), while perceived NSS was negatively associated with SWB (r = −0.269, *p* < 0.001). In addition, perceived PSS was negatively associated with perceived NSS (*r* = −0.318, *p* < 0.001) (Table 1).

Table 5 presents the coefficient estimates from all four models for the regressions of *M* and *Y*. All models controlled for relevant covariates. Full coefficient estimates, including those for covariates, are provided in Supplementary Table S3. Table 4 reports the indices of moderated mediation, and Table 6 presents the indirect effect estimates for all models. The direct association between SFWB and SWB, as hypothesized in H1, was statistically significant across all models (*β* = 0.290, *b* = 0.031, 95% CI [0.028, 0.034]).

**Table 5:** Coefficient estimates for paths from SFWB to SWB via hopelessness.

Predictor	Model 1 (n = 4,308)			Model 2A (n = 4,243)			Model 2B (n = 4,234)			Model 3 (n = 4,234)			Model 4 (n = 4,287)		
	β	b	SE	β	b	SE	β	b	SE	β	b	SE	β	b	SE
M = Hopelessness															
SFWB	−0.36	−0.03***	0.001	−0.31	−0.03***	0.279	−0.31	−0.03***	0.001	−0.29	−0.03***	0.001	−0.30	−0.03***	0.001
NSS				0.17	0.45***	0.040				0.12	0.31***	0.041	0.11	0.30***	0.041
PSS							−0.22	−0.49***	0.032	−0.18	−0.41***	0.033	−0.18	−0.42***	0.034
SFWB × NSS				−0.06	−0.01***	0.003				−0.05	−0.01***	0.003	−0.05	−0.01***	0.003
SFWB × PSS							0.03	0.005*	0.002	0.01	0.00	0.002	−0.00	0.00	0.002
NSS × PSS													−0.03	−0.14**	0.067
SFWB × NSS × PSS													−0.02	−0.01	0.004
	R^2^ = 0.19			R^2^ = 0.22			R^2^ = 0.23			R^2^ = 0.25			R^2^ = 0.25		
	*F* = 100.35***			*F* = 99.93***			*F* = 106.91***			*F* = 99.73***			*F* = 87.61***		
Y = SWB															
SFWB	0.29	0.03***	0.002	0.29	0.03***	0.002	0.29	0.03 ***	0.002	0.29	0.03***	0.002	0.29	0.03***	0.002
Hopelessness	−0.26	−0.32***	0.018	−0.26	−0.32***	0.018	−0.26	−0.32***	0.018	−0.26	−0.32***	0.018	−0.26	−0.32***	0.018
	R^2^ = 0.24			R^2^ = 0.24			R^2^ = 0.24			R^2^ = 0.24			R^2^ = 0.24		
	*F* = 124.53***			*F* = 119.49***			*F* = 119.17***			F = 119.17***			F = 119.17***		

Top panel shows regression results for the mediator (M = hopelessness); bottom panel shows regression results for the outcome (Y = SWB). Coefficient estimates for the other included covariates are provided in Supplementary Table S3. SWB, subjective well-being; SFWB, subjective financial well-being; PSS, positive social support; NSS, negative social support. β = standardized coefficient; b = unstandardized coefficient; SE, standard error; Bootstrap sample size = 10,000. *p < 0.05. **p < 0.01. ***p < 0.001.

**Table 6:** Indirect effects of subjective financial well-being on subjective well-being for models 1-4.

Moderator(s)	Model 1			Model 2A			Model 2B			Model 3			Model 4		
	Effect	SE	95% CI	Effect	SE	95% CI	Effect	SE	95% CI	Effect	SE	95% CI	Effect	SE	95% CI
No Moderator	.093	.001	[.007, .079]												
Low NSS				.007	.001	[.006, .009]									
Moderate NSS				.009	.001	[.007, .010]									
High NSS				.010	.001	[.008, .012]									
Low PSS							.010	.001	[.008, .011]						
Moderate PSS							.009	.001	[.007, .010]						
High PSS							.008	.001	[.007, .009]						
Low NSS, Low PSS										.007	.001	[.005, .009]	.007	.001	[.005, .009]
Low NSS, Moderate PSS										.007	.001	[.005, .008]	.007	.001	[.005, .008]
Low NSS, High PSS										.006	.001	[.005, .008]	.006	.001	[.005, .008]
Moderate NSS, Low PSS										.008	.001	[.007, .010]	.008	.001	[.007, .010]
Moderate NSS, Moderate PSS										.008	.001	[.007, .009]	.008	.001	[.007, .010]
Moderate NSS, High PSS										.008	.001	[.007, .009]	.008	.001	[.007, .010]
High NSS, Low PSS										.010	.001	[.008, .012]	.009	.001	[.007, .011]
High NSS, Moderate PSS										.009	.001	[.008, .011]	.010	.001	[.008, .012]
High NSS, High PSS										.009	.001	[.007, .011]	.010	.001	[.008, .012]

SWB, subjective well-being; SFWB, subjective financial well-being; PSS, positive social support; NSS, negative social support. SE, standard error; Bootstrap sample size = 10,000; Bootstrap 95% CI, confidence interval.

Model 1 examined the mediating role of hopelessness in this association. Hopelessness was negatively associated with SWB (β = −0.259, *b* = −0.315, 95% CI [−0.350, −0.280]) and the indirect effect of SFWB on SWB through hopelessness was statistically significant (β = 0.093, *b* = 0.010, 95% CI [0.008, 0.012], percent mediated = 24.39%), supporting H2. Because both the direct and indirect effects were significant, the mediation was classified as partial. The total effect (i.e., sum of direct and indirect effects) was also statistically significant (β = 0.383, *b* = 0.041, 95% CI [0.038, 0.044]).

**Figure 2:**
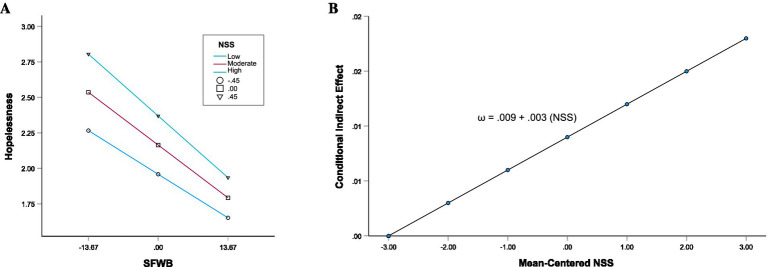
**(A)** Conditional direct effect of SFWB on hopelessness at three levels of NSS. Values represent mean levels of hopelessness; NSS was mean-centered and plotted at the one standard deviation below the mean (−1 SD), at the mean, and one standard deviation above the mean (+1 SD). SFWB, subjective financial well-being; NSS, negative social support. **(B)** Conditional indirect effects of SFWB on SWB. Conditional indirect effect *ω* = a₁ b₁ + a₃ b₁ (NSS), where NSS, negative social support, a₁, a₃, and b₁ are coefficient estimates from Supplementary Equations S3 and S4, estimated in Table 5, Model 2A (see Supplementary Figure S3).

To test H3b-3d, multiple moderated mediation models were estimated (i.e., Models 2A, 2B, 3 and 4). In Model 2A, a moderated mediation model was estimated in which perceived NSS moderated the path from SFWB to hopelessness (Table 5). SFWB was negatively associated with hopelessness (β = −0.309, *b* = −0.027, 95% CI [−0.030, −0.025], *p* < 0.001), and NSS was also significantly associated with hopelessness (β = 0.171, b = 0.435, 95% CI [0.374, 0.531], *p* < 0.001). The interaction term between SFWB and NSS was statistically significant (β = −0.055, *b* = −0.010, 95% CI [−0.016, −0.005], *p* < 0.001), indicating that the association between SFWB and hopelessness depends on levels of NSS (Figure 2A). However, this alone does not confirm moderated mediation. To formally evaluate whether the indirect effect is significantly moderated by NSS, the index of moderated mediation was tested (Hayes, 2015). The result was statistically significant (Index = 0.003, 95% CI [0.002, 0.005]), supporting H3a (Table 4, Model 2A). The conditional indirect effect of SFWB on SWB through hopelessness varied by NSS (Table 6, Model 2A) and can be expressed as *ω* = 0.009 + 0.003 (NSS), where the size of the indirect effect increases with higher levels of NSS (Figure 2B). At low NSS (−0.454), the indirect effect was *b* = 0.007, 95% CI [0.006, 0.009]; at moderate NSS (0), *b* = 0.009, 95% CI [0.007, 0.010]; and at high NSS (+0.454), *b* = 0.010, 95% CI [0.008, 0.012]. Thus, the indirect effect increased with higher levels of NSS (Effect _indirect_ = 0.007 to 0.010).

**Figure 3:**
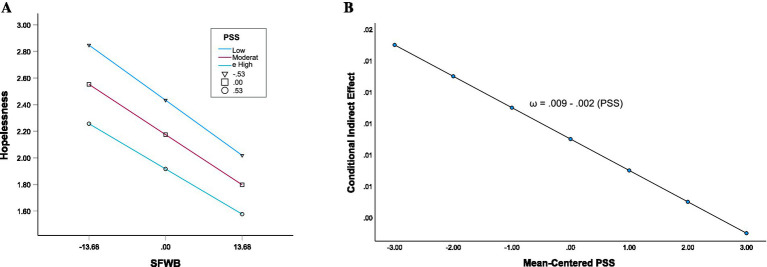
**(A)** Conditional direct effect of SFWB on hopelessness at three levels of PSS. Values represent mean levels of hopelessness; PSS was mean-centered and plotted at one standard deviation below the mean (−1 *SD*), at the mean, and one standard deviation above the mean (+1 *SD*). SFWB, subjective financial well-being; PSS, positive social support. **(B)** Conditional indirect effects of SFWB on SWB. The conditional indirect effect is calculated as *ω* = *a₁b₁ + a₃b₁(PSS)*, where PSS, positive social support*. a₁, a₃,* and *b₁* are coefficient estimates from Supplementary Equations S3 and S4, estimated in Table 5, Model 2B (see Supplementary Figure S3).

In Model 2B (Table 5), a moderated mediation model was estimated in which perceived PSS moderated the path from SFWB to hopelessness. SFWB was negatively associated with hopelessness (β = −0.314, *b* = −0.028, 95% CI [−0.030, −0.025], *p* < 0.001), and PSS was also significantly associated with hopelessness (β = −0.215, *b* = −0.490, 95% CI [−0.552, −0.428], p < 0.001). The interaction between SFWB and PSS was statistically significant (β = 0.032, *b* = 0.005, 95% CI [0.001, 0.009], *p* = 0.018), indicating that the strength of the association between SFWB and hopelessness varies as a function of PSS (Figure 3A). The standardized interaction between SFWB and NSS (β = −0.055) was stronger in magnitude than the interaction between SFWB and PSS (β = 0.032), suggesting that NSS had a greater moderating effect on the SFWB–hopelessness association (Figures 2A, 3A). The index of moderated mediation was statistically significant (Index = −0.002, 95% CI [−0.003, 0.000]), supporting H3b that PSS moderates the indirect effect (Table 4 Model 2B). The conditional indirect effects of SFWB on SWB through hopelessness varied by PSS (Table 6, Model 2B) and can be expressed as ω = 0.009–0.002 (PSS), where the size of the indirect effect decreases with higher levels of PSS (Figure 3B). At low PSS (−0.528), the indirect effect was *b* = 0.010, 95% CI [0.008, 0.011]; at moderate PSS (0), *b* = 0.009, 95% CI [0.007, 0.010]; and at high PSS (+0.528), *b* = 0.008, 95% CI [0.007, 0.009]. Thus, the indirect effect decreased slightly as PSS increased (Effect _indirect_ = 0.010 to 0.008).

These findings are consistent with H3a–3b and indicate that while both forms of perceived social support are relevant to the indirect association between SFWB and SWB, negative support appears to exert a comparatively stronger influence. However, to further evaluate their relative influence (i.e., H3c–3d), both PSS and NSS were included simultaneously within the same model (Model 3 and 4).

**Figure 4:**
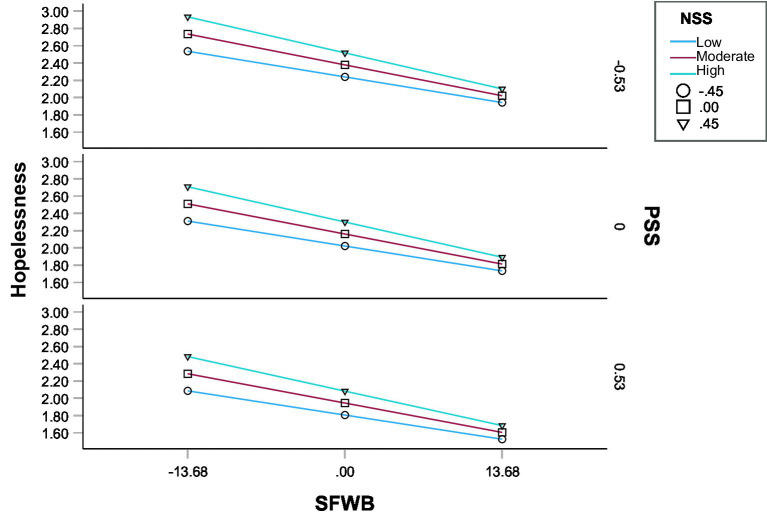
Conditional direct effect of SFWB on hopelessness in Model 3. Values represent mean levels of hopelessness; NSS and PSS were mean-centered and plotted at one standard deviation below the mean (−1 *SD*), at the mean, and one standard deviation above the mean (+1 *SD*). SFWB, subjective financial well-being; NSS, negative social support; PSS, positive social support.

Model 3 examined the simultaneous moderating effects of NSS and PSS on the relationship between SFWB and hopelessness. Unlike Models 2A and 2B, which tested each moderator separately, Model 3 entered both moderators simultaneously to estimate their independent effects without assuming interaction between NSS and PSS (Hayes, 2017). The association between SFWB and hopelessness remained significant and negative (β = −0.290, *b* = −0.025, 95% CI [−0.028, −0.023], *p* < 0.001) after controlling for both moderators (Figure 4). NSS was positively associated with hopelessness (β = 0.259, *b* = 0.306, 95% CI [0.226, 0.387], *p* < 0.001), whereas PSS was negatively associated with hopelessness (β = −0.281, *b* = −0.411, 95% CI [−0.475, −0.347], *p* < 0.001). The SFWB × NSS interaction was statistically significant (β = −0.051, *b* = −0.010, 95% CI [−0.015, −0.004], *p* < 0.001), indicating that the inverse SFWB–hopelessness association was stronger at higher NSS. The SFWB × PSS interaction was small and nonsignificant (β = 0.007, *b* = 0.001, 95% CI [−0.003, −0.006], *p* = 0.606), suggesting that PSS did not significantly moderate the path from SFWB to hopelessness in the presence of NSS. Conditional indirect effects of SFWB on SWB were examined at low, moderate, and high levels of NSS (−0.454, 0, +0.454) and at low, moderate, high levels of PSS (−0.528, 0, +0.528). The conditional indirect effects were statistically significant at all combinations of NSS and PSS tested (Table 6, Model 3).

Across moderator values, the indirect effect ranged from 0.006 (95% CI [0.005, 0.008]) at low NSS and high PSS to 0.010 (95% CI [0.008, 0.012]) at high NSS and low PSS, indicating that the mediated relationship was present under all conditions (Effect _indirect_ = 0.006 to 0.010). The index of moderated mediation for NSS was significant (index = 0.003, 95% CI [0.001, 0.005]), suggesting that higher levels of NSS amplified the indirect effect of SFWB on SWB through hopelessness. In contrast, the index for PSS was nonsignificant (index = 0.000, 95% CI [−0.002, 0.001]), indicating that PSS did not independently moderate the mediation pathway (Table 4, Model 3). These findings suggest that while hopelessness serves as a robust mediator across social support contexts, the strength of this mediation is particularly sensitive to variations in NSS. Practically, this means that individuals experiencing greater NSS may be more vulnerable to the adverse impact of low SFWB on hopelessness, which in turn undermines their overall SWB, thereby supporting H3c.

**Figure 5:**
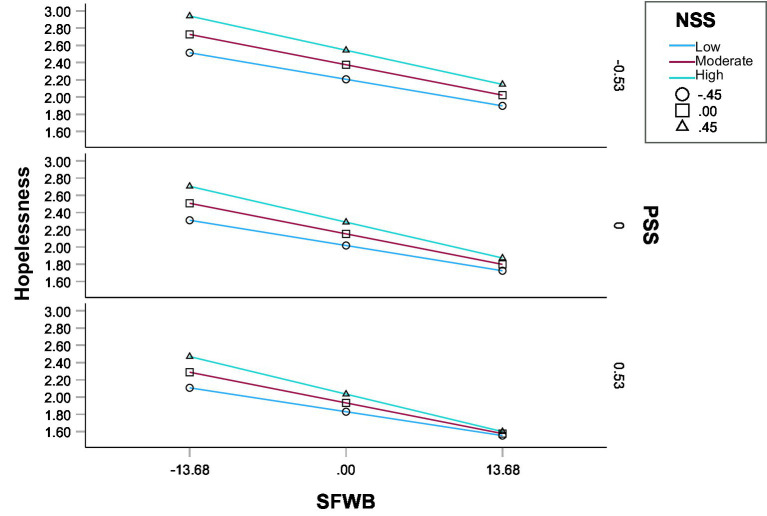
Conditional direct effect of SFWB on hopelessness in Model 4. Values represent mean levels of hopelessness; NSS and PSS were mean-centered and plotted at one standard deviation below the mean (−1 *SD*), at the mean, and one standard deviation above the mean (+1 *SD*). SFWB, subjective financial well-being; NSS, negative social support; PSS, positive social support.

To test H3d, Model 4 examined whether the indirect effect of SFWB on SWB was jointly moderated by NSS and PSS, including their interaction—i.e., a moderated-moderated mediation. Specifically, the model tested whether the moderation of the indirect effect by NSS varied depending on the level of PSS. SFWB was negatively associated with hopelessness (β = −0.295, *b* = −0.026, *p* < 0.001, 95% CI [−0.029, −0.023]). NSS was positively related to hopelessness (β = 0.113, *b* = 0.299, 95% CI [0.218, 0.380], *p* < 0.001), whereas PSS was negatively related to hopelessness (β = −0.184, *b* = −0.419, 95% CI [−0.486, −0.353], *p* < 0.001). The SFWB × NSS interaction was significant (β = −0.053, *b* = −0.010, 95% CI [−0.016, −0.004], *p* < 0.001), suggesting that the negative association between SFWB and hopelessness was stronger at higher levels of NSS. The NSS × PSS interaction was also significant (β = −0.031, *b* = −0.139, 95% CI [−0.271, −0.007], *p* = 0.015), indicating that the effect of NSS on hopelessness was more pronounced at lower levels of PSS (Figure 5). Neither the SFWB × PSS interaction nor the three-way SFWB × NSS × PSS interaction was significant.

Controlling for SFWB, hopelessness was negatively associated with SWB (β = −0.242, *b* = −0.260, *p* < 0.001, 95% CI [−0.352, −0.281]). Bootstrapping (10,000 samples) indicated that the indirect effect of SFWB on SWB through hopelessness was significant across all combinations of NSS and PSS (all 95% CIs excluded zero), ranging from 0.006 to 0.010 (Table 6, Model 4). The index of moderated-moderated mediation was nonsignificant (index = 0.002, 95% CI [−0.001, 0.005]), indicating no evidence that the indirect effect depended jointly on NSS and PSS. However, the indices of conditional moderated mediation by NSS were significant at all levels of PSS (low PSS: index = 0.002, 95% CI [0.000, 0.005]; moderate PSS: index = 0.003, 95% CI [0.001, 0.005]; high PSS: index = 0.004, 95% CI [0.002, 0.007]), suggesting that higher NSS consistently strengthened the indirect effect of SFWB on SWB through hopelessness, regardless of PSS level (Table 4, Model 4). These findings indicate that hopelessness mediates the relationship between SFWB and SWB and that this mediation is amplified by higher levels of NSS, irrespective of PSS. The hypothesized moderated-moderated mediation was not supported; however, the significant indices of conditional moderated mediation by NSS support the conclusion that NSS intensifies the indirect pathway, consistent with the broader theoretical expectation that adverse social environments exacerbate the psychological impact of financial strain.

## 7 Discussion

The present study examined moderated mediation models linking SFWB with SWB, with hopelessness as a potential mediator and perceived social support as a moderator among adults aged 50 and older in the United States. A key strength of this study is the use of a multidimensional measure of financial perceptions. Unlike single-item financial satisfaction indicators or objective metrics such as income, the CFPB financial well-being scale provides a comprehensive assessment of SFWB, capturing both present security (e.g., ability to meet expenses, resilience to shocks) and future outlook (e.g., confidence in sustaining financial needs, freedom of choice). Developed using item response theory and normed on large national samples with age-specific scoring tables, the scale ensures reliable and comparable measurement across groups (Brüggen et al., 2017; Consumer Financial Protection Bureau, 2017). In addition, this study extends prior research by examining both positive and negative dimensions of social support, enabling the evaluation of their independent and combined effects on the pathways linking financial perceptions, hopelessness, and well-being.

### 7.1 Summary of findings

Three main aims guided this study. Aim 1 tested whether SFWB is directly associated with SWB. Aim 2 examined whether hopelessness mediates the SFWB-SWB association. Aim 3 evaluated whether perceived social support, both positive and negative, moderates the indirect association between SFWB and SWB through hopelessness.

Findings for Aim 1 underscored the positive association between SFWB and SWB in all models. This result is consistent with prior work showing that subjective evaluations of one’s financial condition are strongly linked to life satisfaction and psychological adjustment in later life, independent of objective financial resources (Yeo and Lee, 2019).

Findings for Aim 2 indicated that hopelessness explained about one-quarter of the link between SFWB and SWB. This suggests that emotional distress partly accounts for this relationship, as financial strain can increase worry and hopelessness (Frankham et al., 2020), lowering life satisfaction. Both the total and direct effects remained significant, indicating partial mediation.

Findings for Aim 3 supported that the indirect effect of SFWB on SWB varied systematically based on levels of NSS and PSS. When examined separately, both NSS and PSS moderated the SFWB-SWB pathway, but with slightly different effect size. Higher NSS amplified the pathway, whereas PSS exerted only a modest buffering effect, slightly weakening the association as positive support increased. This result is consistent with prior findings suggesting that negative social exchanges often exert a stronger influence on older adults’ psychological health than positive exchanges, even though supportive interactions can promote resilience and well-being (Newsom et al., 2005; Rook, 2001; Rook, 2015). When NSS and PSS were modeled as independent moderators, only NSS significantly influenced the link between SFWB and hopelessness. The conditional indirect effects were stronger with higher levels of negative support, and weakened as negative support decreased and positive support increased. A separate model examined whether the potential interdependence of NSS and PSS jointly influenced the SFWB-hopelessness link, as well as the indirect pathway. This included a three-way interaction term (SFWB × NSS × PSS) to assess whether the effect of negative support varied as a function of positive support. Results indicated that the interaction was not statistically significant, suggesting that negative social support intensified the SFWB–hopelessness association regardless of the level of positive support.

### 7.2 Interpretations

The consistent association between perceived financial satisfaction and life satisfaction underscores the psychological importance of financial perceptions in later life. Beyond material resources, older adults’ sense of financial security and control plays a central role in shaping emotional well-being and life satisfaction. These findings align with prior evidence emphasizing the multidimensional nature of FWB, which integrates cognitive appraisals and affective experiences of security (Riitsalu et al., 2024).

The partial mediation suggests that while hopelessness plays a central role in linking financial perceptions to well-being, other mechanisms (e.g., perceived control, optimism, or self-esteem) may also contribute to this pathway (Dumitrache et al., 2015). Lower financial satisfaction likely reflects broader psychological vulnerability, encompassing factors such as low morale, depressive symptoms, and reduced self-esteem (Angel et al., 2003). Prior evidence shows that older adults experiencing depression incur significantly higher healthcare costs (Katon et al., 2003), which may further erode both financial satisfaction and life satisfaction. These findings underscore the importance of considering broader social, contextual, and psychological factors (e.g., depression) when examining the emotional consequences of financial strain in later life.

The moderated mediation analyses suggest that the psychological impact of financial perceptions depends meaningfully on the quality of social interactions. When examined separately, both negative and positive social support shaped the indirect link between financial perceptions and overall well-being through hopelessness, though in opposite ways. Negative exchanges tended to intensify the emotional burden of financial insecurity and control, whereas positive support offered only limited protection. This pattern suggests that conflict, criticism, or strain in relationships may amplify the distress associated with financial strain, while supportive interactions provide a modest but insufficient buffer. When negative and positive support were considered together as independent moderators, only negative exchanges continued to exert a significant influence, indicating that the harmful effects of negative interactions outweigh the benefits of positive ones (Rook, 2015; Lincoln, 2000). Moreover, when both were modeled as interdependent—that is, when the influence of one depended on the other—the joint effect was nonsignificant. This finding implies that the presence of positive support does not neutralize the emotional costs of negative interactions. Overall, these results underscore a consistent asymmetry in social relationships: negative exchanges magnify the psychological impact of financial insecurity and control more strongly than positive ones can mitigate it, reflecting the well-established “negativity effect” in social functioning during later life.

### 7.3 Practical implications

The practical implication of this study emphasizes that supporting older adults’ well-being requires interventions that go beyond finances alone. Financial interventions such as financial education and counseling tailored to aging populations can reduce insecurity and improve confidence in daily decision-making. These programs may also include housing assistance or financial planning resources that enhance a sense of control and stability in later life. Beyond improving financial literacy, such interventions can foster a stronger sense of autonomy and reduce the psychological burden associated with financial uncertainty. Community-based programs that integrate personalized guidance, for example helping older adults navigate benefit eligibility, debt management, or healthcare expenses, can further strengthen financial resilience. In doing so, these initiatives not only address immediate economic concerns but also contribute to a broader sense of empowerment and well-being in later life.

Psychosocial interventions are equally important. Efforts to minimize negative social interactions, such as family conflict resolution or peer-support programs, may buffer the emotional costs of financial stress. Prior research shows that financial security promotes health in part by enabling social connectedness (Cruwys et al., 2019), underscoring the need to consider both social support and financial satisfaction together. In addition, perceived social support or isolation has been shown to influence mental health, which in turn shapes physical health and overall well-being (Rohde and D'Ambrosio, 2014), and subjective financial situation (Doanward et al., 2020). Implementing programs that reduce isolation and strengthen supportive networks should therefore be a policy priority. Integrating these approaches with accessible mental health services offers a concrete pathway to reduce hopelessness and promote resilience in later life.

## 8 Limitations/future direction

It is important to acknowledge several limitations of the present study. First, the cross-sectional design limits causal inference. Second, reliance on self-reported measures introduces potential common-method bias. Third, the sample context may limit generalizability to other populations of midlife and older adulthood. Finally, beyond social support, individual characteristics such as personality traits or coping styles may interact with financial perceptions and hopelessness and should be considered in future research. Longitudinal and cross-cultural studies are needed to establish causal mechanisms and generalize findings beyond U.S. older adults.

Despite these limitations, the study advances existing research by illustrating how financial perceptions, emotional processes, and social contexts jointly shape well-being in later life. Future research should employ longitudinal designs and examine additional mediators, including perceived control and self-efficacy, to clarify the mechanisms through which financial perceptions influence quality of life in aging populations.

## 9 Conclusion

For older adults, the subjective evaluation of FWB directly shapes financial autonomy, security, and overall life satisfaction. Individuals with lower SFWB were more likely to experience increased feelings of hopelessness, which in turn were associated with lower SWB. Additionally, negative social support is a stronger risk factor than positive social support. To enhance the overall well-being of older adults, interventions should address both financial and psychological aspects. Cross-sector policies that provide financial education, social support, and age-friendly initiatives such as affordable housing are needed for improved well-being in later life. Finally, collaboration across financial, mental health, and social work professionals, alongside ongoing research, is essential to provide comprehensive and effective support for older adults.

## Data Availability

The datasets analyzed for this study can be found in the Health and Retirement Study at https://hrsdata.isr.umich.edu/data-products/2020-hrs-core and https://hrsdata.isr.umich.edu/data-products/rand-hrs-longitudinal-file-2022. Further inquiries can be directed to the corresponding author.

## Appendices

### Appendix A. Subjective well-being

Please say how much you agree or disagree with the following statements.

1. In most ways my life is close to ideal.
2. The conditions of my life are excellent.
3. I am satisfied with my life.
4. So far, I have gotten the important things I want in life.
5. If I could live my life again, I would change almost nothing.

1 = Strongly disagree, 2 = Somewhat disagree, 3 = Slightly disagree, 4 = Neither agree nor disagree, 5 = Slightly agree, 6 = Somewhat agree, 7 = Strongly agree.

### Appendix B. Hopelessness

Please say how much you agree or disagree with the following statements:

1. I feel it is impossible for me to reach the goals that I would like to strive for.
2. The future seems hopeless to me and I cannot believe that things are changing for the better.
3. I do not expect to get what I really want.
4. There’s no use in really trying to get something I want because I probably will not get it.

1 = Strongly disagree, 2 = Somewhat disagree, 3 = Slightly disagree, 4 = Slightly agree, 5 = Somewhat agree, 6 = Strongly agree.

### Appendix C. Perceived social support

Please check the answer which best shows how you feel about each statement.

#### Positive social support

For each relationship category: spouses, children, family, and friends

1. How much do they really understand the way you feel about things?
2. How much can you rely on them if you have a serious problem?
3. How much can you open up to them if you need to talk about your worries?

#### Negative social support

For each relationship category: spouses, children, family, and friends

1. How often do they make too many demands on you?
2. How much do they criticize you?
3. How much do they let you down when you are counting on them?
4. How much do they get on your nerves?

1 = A lot, 2 = Some, 3 = A little, 4 = Not at all.
