# Supplementary material for: Financial perceptions and subjective well-being among older adults
Source: Front Psychol. 2026 Jan 6;16:1709795. doi: 10.3389/fpsyg.2025.1709795 (PMC12816292; doi:10.3389/fpsyg.2025.1709795)
Supplement: Supplementary file 1 [file Supplementary_file_1.docx]

**Supplementary Materials for Financial Perceptions and Subjective Well-being Among Older Adults**

1. **Predicting Subjective Financial Well-Being, adapted from Consumer Financial Protection Bureau (CFPB)**

***Stata module***

The Stata program **pfwb.ado**, developed by Nicholas (2017), was used to predict financial well-being (PFWB) scale scores based on the Consumer Financial Protection Bureau (Consumer Financial Protection Bureau, 2017) survey instrument. The corresponding help file is also available from the same source. Both files can be accessed at <https://econpapers.repec.org/software/bocbocode/s458353.htm>. The module was installed within Stata using the command: ssc install pfwb.

1. **PROCESS macro in SPSS Statistics (version 29)**

PROCESS is a computational tool invented by [Canadian Centre for Research Analysis and Methods (CCRAM)](https://haskayne.ucalgary.ca/CCRAM) expert Andrew F. Hayes. It is freely-available for SPSS, SAS, and R, and has become widely used throughout the behavioral sciences as well as in business research, medicine, and public health for easing the estimation of effects in mediation, moderation, and conditional process analysis . In this study, the PROCESS v5.0 macro for SPSS was installed using the guide and package available at <https://haskayne.ucalgary.ca/CCRAM/resource-hub>.

1. **Detailed Explanation for Sample Size**

The data for this study were drawn from the 2020 wave of the Health and Retirement Study (HRS), a nationally representative longitudinal survey of adults over the age of 50 in the United States. Since 2006, HRS has administered a psychosocial and lifestyle self-administered questionnaire (SAQ), known as the “Leave-Behind” (LB) questionnaire, to a randomly selected 50% subsample of respondents who completed the enhanced face-to-face interview. This questionnaire collects data on subjective well-being, psychosocial resources, and lifestyle characteristics. The LB instrument is distributed biennially and returned by mail.

In 2020, 15,723 respondents participated in the HRS Core interview. Of those, 4,629 completed and returned the LB questionnaire by mail. Cases were excluded if the LB questionnaire was completed by someone other than the assigned respondent (n = 59). Although HRS primarily targets individuals aged 50 and older, younger spouses are sometimes included; 50 participants under the age of 50 were excluded from the analytic sample. **Figure S1** illustrates the sample selection process.

1. **Covariates**

We used the RAND HRS Longitudinal File 2022 (Version 1) (RAND Center for the Study of Aging, 2025; Health and Retirement Study, 2025) to obtain covariate information. The RAND file provides a user-friendly, cleaned, and processed version of a subset of the original HRS data, including most individual-level variables. Details on the covariates and any recoding procedures are presented in **Table S1**.

1. **Missing Data Handling**

All analyses were conducted using the PROCESS macro in *SPSS*, which applies listwise deletion by default. Prior to estimation, the dataset was screened for missingness. **Table S2** presents the extent of missing data across the study variables.

1. **Statistical Analysis**

***Mediation (Model 1)***

**Figure S2** shows the study’s conceptual and statistical frameworks for the mediation analysis. From the equations S1 and S2, the indirect effect is *a_1_ b_1_* and the direct effect is c'.

$\boldsymbol{M}=i_{M}+a_{1}\boldsymbol{X}+\sum_{j=1}^{7} a_{j+1}U_{j}+e_{M}$ $\left( S1 \right)$

$\boldsymbol{Y}=i_{Y}+c^{'}\boldsymbol{X}+b_{1}\boldsymbol{M}+\sum_{j=1}^{7} b_{j+1}U_{j}+e_{Y} (S2)$

***Moderated Mediation (Model 2)***

**Figure S3** shows the study’s conceptual and statistical frameworks for the moderated mediation analysis with a single moderator. From the equations S3 and S4, the conditional indirect effect of is *(a1 + a3W)b1*, where *W* is the moderator (either NSS or PSS) and the direct effect is c'.

$\boldsymbol{M}=i_{M}+a_{1}\boldsymbol{X}+a_{2}\boldsymbol{W}+a_{3}\boldsymbol{XW}+\sum_{j=1}^{7} a_{j+3}U_{j}+e_{M} \left( S3 \right)$

$\boldsymbol{Y}=i_{Y}+c^{'}\boldsymbol{X}+b_{1}\boldsymbol{M}+\sum_{j=1}^{7} b_{j+1}U_{j}+e_{Y} (S4)$

***Partial Moderated Mediation (Model 3)***

**Figure S4** shows the study’s conceptual and statistical frameworks for the partial moderated mediation analysis with two independent moderators. From the equations S5 and S6, the conditional indirect effect of is *a_1_b_1_ + a_4_b_1_W + a_5_b_1_Z*, where *W* and *Z*represent NSS and PSS, respectively, and the direct effect is c'.

$\boldsymbol{M=}i_{M}\boldsymbol{+}a_{1}\boldsymbol{X+}a_{2}\boldsymbol{W+}a_{3}\boldsymbol{Z+}a_{4}\boldsymbol{XW+}a_{5}\boldsymbol{XZ+}\sum_{j=1}^{7} a_{j+5}U_{j}+ e_{M} (S5)$

$\boldsymbol{Y}=i_{Y}+c^{'}\boldsymbol{X}+b_{1}\boldsymbol{M}+\sum_{j=1}^{7} b_{j+1}U_{j}+e_{Y} (S6)$

**Table S1.** Detailed covariates and reassigned categories.

| Covariates | n | Combined category |
| --- | --- | --- |
| Age | | |
| Age 50-61 | 1,193 |  |
| Age ≥62 | 3,376 |  |
| Gender | | |
| Female | 2,752 |  |
| Male | 1,818 |  |
| Race | | |
| White/Caucasian | 3,244 |  |
| Black/African American | 875 |  |
| Other ^a^ | 433 |  |
| Ethnicity | | |
| Hispanic | 3,927 |  |
| Not Hispanic | 639 |  |
| Marital status | | |
| Married | 2,590 | Combined in married: 2,832 |
| Married, spouse absent | 13 |  |
| Partnered | 22 |  |
| Separated | 57 | Combined in not married: 1,723 |
| Divorced | 614 |  |
| Widowed | 796 |  |
| Never married | 256 |  |
| Education level | | |
| > High school | 564 | Combined in high school/GED or less than high school: 2,013 |
| GED | 232 |  |
| High school graduate | 1,217 |  |
| Some college | 1,238 |  |
| College and above | 1,319 |  |
| Household Poverty threshold | | |
| Below | 484 |  |
| Above | 4,058 |  |

This table presents the original response categories for each variable and the corresponding recoded categories used in the analysis.

^a^ Other includes American Indian, Alaskan Native, Asian, Native Hawaiian, and Pacific Islander

**Table S2***.* Missing values (N=4,570).

| Variables | No. missing | n ^a^ |
| --- | --- | --- |
| SFWB | 111 | 4,459 |
| SWB | 64 | 4,506 |
| Hopelessness | 63 | 4,507 |
| PSS | 101 | 4,469 |
| NSS | 91 | 4,479 |
| Age | 1 | 4,569 |
| Race | 18 | 4,552 |
| Ethnicity | 4 | 4,566 |
| Marital status | 15 | 4,555 |
| Education level | 0 | 4,570 |
| Poverty level | 28 | 4,542 |

SFWB = subjective financial well-being; SWB = subjective well-being; PSS = positive social support; NSS = negative social support

^a^ Of the 4,570 participants, 4,234 had complete data for all variables included in the analysis.

**Table S3***.* Coefficient estimates for paths from SFWB to SWB via hopelessness.

|  | Model 1  (*n* = 4,308) | | | | Model 2A  (*n* = 4,243) | | | Model 2B  (*n* = 4,234) | | | Model 3  (*n* = 4,234) | | | Model 4  (*n* = 4,287) | | |
| --- | --- | --- | --- | --- | --- | --- | --- | --- | --- | --- | --- | --- | --- | --- | --- | --- |
| Predictor | β | b | | SE | β | b | SE | β | b | SE | β | b | SE | β | b | SE |
| ***M* = Hopelessness** | | | | | | | | | | | | | | | | |
| SFWB | –.36 | | –.03*** | .001 | –.31 | –.03*** | .279 | –.31 | –.03*** | .001 | –.29 | –.03*** | .001 | –.30 | –.03*** | .001 |
| NSS |  | |  |  | .17 | .45*** | .040 |  |  |  | .12 | .31*** | .041 | .11 | .30*** | .041 |
| PSS |  | |  |  |  |  |  | –.22 | –.49*** | .032 | –.18 | –.41*** | .033 | –.18 | –.42*** | .034 |
| SFWB × NSS |  | |  |  | –.06 | –.01*** | .003 |  |  |  | –.05 | –.01*** | .003 | –.05 | –.01*** | .003 |
| SFWB × PSS |  | |  |  |  |  |  | .03 | .01* | .002 | .01 | .00 | .002 | –.00 | .00 | .002 |
| NSS × PSS |  | |  |  |  |  |  |  |  |  |  |  |  | –.03 | –.14** | .067 |
| SFWB × NSS × PSS |  | |  |  |  |  |  |  |  |  |  |  |  | –.02 | –.01 | .004 |
| Age 50-61 | −.08 | | −.21*** | .039 | –.09 | –.25*** | .039 | –.08 | –.21*** | .038 | –.08 | –.24*** | .038 | –.09 | –.24*** | .038 |
| Female | −.07 | | −.16*** | .035 | –.07 | –.17*** | .034 | –.04 | –.09** | .034 | –.04 | –.11** | .034 | –.05 | –.11*** | .034 |
| African American/Black | −.08 | | −.24*** | .045 | –.09 | –.27*** | .044 | –.07 | –.22*** | .044 | –.08 | –.24*** | .043 | –.08 | –.24*** | .043 |
| Race Other ^a^ | .01 | | .03 | .063 | .00 | .00 | .062 | .01 | .02 | .061 | –.00 | –.01 | .061 | –.00 | –.01 | .061 |
| Hispanic | .01 | | .02 | .055 | .00 | .00 | .054 | .02 | .07 | .053 | .01 | .05 | .053 | .01 | .05 | .053 |
| Married | −.02 | | −.04 | .037 | –.03 | –.08* | .037 | –.01 | –.02 | .036 | –.02 | –.06 | .036 | –.02 | –.06 | .036 |
| Some college | −.11 | | −.30*** | .041 | –.11 | –.30*** | .041 | –.11 | –.29*** | .040 | –.11 | –.30*** | .040 | –.11 | –.30*** | .040 |
| College Degree/ above | −.17 | | −.45*** | .042 | –.18 | –.47*** | .041 | –.18 | –.44*** | .041 | –.17 | –.46*** | .041 | –.17 | –.46*** | .041 |
| Below poverty threshold | .05 | | .21*** | .059 | .04 | .18** | .059 | .05 | .22*** | .058 | .05 | .19*** | .058 | .05 | .19*** | .058 |
|  | *R²* = .19 | | |  | *R²* =.22 | |  | *R²* =.23 | |  | *R²* =.25 | |  | *R²* =.25 | |  |
|  | *F* = 100.35*** | | |  | *F* = 99.93*** | |  | *F* = 106.910*** | |  | *F =* 99.726*** | |  | *F* = 87.609*** | |  |
| ***Y* = SWB** | | | | | | | | | | | | | | | | |
| SFWB | .29 | .03*** | | .002 | .29 | .03*** | .002 | .29 | .03*** | .002 | .29 | .03*** | .002 | .29 | .03*** | .002 |
| Hopelessness | –.26 | –.32*** | | .018 | –.26 | –.32*** | .018 | –.26 | –.32*** | .018 | –.26 | –.32*** | .018 | –.26 | –.32*** | .018 |
| Age 50-61 | –.04 | –.12** | | .046 | –.04 | –.12** | .047 | –.04 | –.12** | .047 | –.04 | –.12** | .047 | –.04 | –.12** | .047 |
| Female | .01 | .03 | | .041 | .01 | .04 | .041 | .01 | .04 | .041 | .01 | .04 | .041 | .01 | .04 | .041 |
| African American/Black | –.04 | –.15** | | .053 | –.04 | –.14** | .053 | –.04 | –.14** | .053 | –.04 | –.14** | .053 | –.04 | –.14** | .053 |
| Race Other ^a^ | .04 | .20** | | .074 | .04 | .19** | .074 | .04 | .19** | .075 | .04 | .19** | .075 | .04 | .19** | .075 |
| Hispanic | .08 | .33*** | | .064 | .08 | .32*** | .065 | .08 | .33*** | .065 | .08 | .33*** | .065 | .08 | .33*** | .065 |
| Married | .12 | .37*** | | .043 | .12 | .37*** | .044 | .12 | .37*** | .044 | .13 | .37*** | .044 | .12 | .37*** | .044 |
| Some college | –.04 | –.12* | | .049 | –.04 | –.12* | .049 | –.04 | –.12* | .049 | –.04 | –.12* | .049 | –.04 | –.12** | .049 |
| College Degree/above | –.04 | –.12* | | .050 | –.04 | –.12* | .050 | –.04 | –.12* | .050 | –.04 | –.12* | .050 | –.04 | –.12* | .050 |
| Below poverty threshold | .00 | –.00 | | .069 | –.00 | –.01 | .070 | –.00 | –.00 | .071 | –.00 | –.00 | .071 | –.00 | –.00 | .071 |
|  | *R²*= .24 | | |  | *R²* = .24 | |  | *R²*= .24 | |  | *R²*= .24 | |  | *R²* = .24 | |  |
|  | *F*  = 124.53*** | | |  | *F*  = 119.492*** | |  | *F* = 119.165*** | |  | *F* = 119.165*** | |  | *F* = 119.165*** | |  |

Coefficient estimates are presented from two regressions: one for the mediator (*M* = hopelessness) and one for the outcome (*Y* = SWB). The top panel displays results for the mediator, and the bottom panel displays results for the outcome.

The reference groups are age ≥ 62 years; male; White/Caucasian; non-Hispanic; not married; high school diploma/GED or less; and above the poverty threshold.

SWB = subjective well-being; SFWB = subjective financial well-being; PSS = positive social support; NSS = negative social support.

β = standardized coefficient; *b* = unstandardized coefficient; *SE* = standard error; Bootstrap sample size= 10,000

^a^ Other includes American Indian, Alaskan Native, Asian, Native Hawaiian, and Pacific Islander.

**p < .05. **p < .01. ***p < .001.*

**Figure S1***.* Flow diagram of the sample selection.

**15,723** Respondents in 2020

**11,094** excluded (did not complete leave behind (LB) questionnaire)

**4,629** completed LB

**59** excluded (age < 50)

**4,570** participants

**Figure S2***.* Mediation model of associations between SFWB and SWB in conceptual and statistical forms.

| **A.**  Conceptual Diagram | **B.** Statistical Diagram  *e_M_* |
| --- | --- |
| Hopelessness  SFWB  SFWB  SWB | *e_Y_*  Hopelessness  *c^'^*  *a_1_*  *b_1_*  SWB |

This figure illustrates Model 1; Panel A displays hypothesized associations, with hopelessness serving as the mediator between SFWB and SWB; Panel B presents the coefficient estimates for associations among SFWB, SWB, and hopelessness. For simplicity, other covariates included in the model are not shown; *e_M_* and *e_Y_* are error terms.

Direct effect = *c^’^*; Conditional indirect effect of SFWB on SWB through hopelessness = *a_1_b_1_.*

SFWB = subjective financial well-being; SWB = subjective well-being.

**Figure S3**. A First stage moderated mediation model of associations between SFWB and SWB in conceptual and statistical forms.

| **A.**  Conceptual Diagram | **B.** Statistical Diagram  *e_M_* | |
| --- | --- | --- |
| SFWB  *a_1_*  Hopelessness  NSS  SFWB  SWB | | *c^'^*  *e_Y_*  *b_1_*  Hopelessness  SWB  *a_2_*  *a_3_*  SFWB $\times$NSS  NSS |

This figure illustrates Model 2A, where NSS is the single moderator. Panel A displays hypothesized associations, with hopelessness serving as the mediator between SFWB and SWB, and NSS serving as the moderator. Panel B presents the coefficient estimates for associations among SFWB, SWB, hopelessness, and NSS. For simplicity, other covariates included in the model are not shown; *e _M_* and *e _Y_* are error terms. The model for PSS (Model 2B) as a single moderator is specified analogously.

Direct effect = *c^’^*; Conditional indirect effect of SFWB on SWB through hopelessness = [*a_1_* + *a_3_* (NSS)] *b_1_*.

SFWB = subjective financial well-being; SWB = subjective well-being; NSS = negative social support.

**Figure S4** *.* A First stage moderated mediation model of associations between SFWB and SWB in conceptual and statistical forms.

| **A.**  Conceptual Diagram | **B.** Statistical Diagram | |
| --- | --- | --- |
| *a_1_*  SFWB  NSS  Hopelessness  PSS  NSS  SFWB  SWB | | *a_4_*  *a_3_*  *a_5_*  *b_1_*  c^'^  SWB  Hopelessness  *e_Y_*  *e_M_*  PSS  SFWB $\times$NSS  SFWB $\times$PSS  *a_2_* |

This figure illustrates Model 3. Panel A displays hypothesized associations, with hopelessness serving as the mediator between SFWB and SWB and NSS and PSS serving as the moderators. Panel B presents the coefficient estimates for associations among SFWB, SWB, hopelessness, NSS and PSS. For simplicity, other covariates included in the model are not shown; *e_M_* and *e_Y_* are error terms.

Direct effect = *c^’^*; Conditional indirect effect of SFWB on SWB through hopelessness = [*a_1_* + *a_4_* (NSS) + *a_5_* (PSS)]*b_1_*.

SFWB = subjective financial well-being; SWB = subjective well-being; NSS = negative social support; PSS = positive social support.

# **References**

Consumer Financial Protection Bureau. (2017). *CFPB Financial Well-Being Scale: Scale development technical report.* Consumer Financial Protection Bureau. <https://sjdm.org/dmidi/files/CFPB_Financial_Well-Being_Scale_Technical_Report.pdf>

Hayes, A. F. (2017). Partial, conditional, and moderated moderated mediation: Quantification, inference, and interpretation. *Communication Monographs, 85*(1), 4-40. <https://doi.org/10.1080/03637751.2017.1352100>

Health and Retirement Study. (2025). *(RAND HRS Longitudinal File 2022 (V1)) public use dataset.* Ann Arbor, MI: Produced and distributed by the University of Michigan with funding from the National Institute on Aging (grant numbers NIA U01AG009740 and NIA R01AG073289).

Nicholas, A. (2017). PFWB: Stata module to predict Financial Well-Being scale scores from CFPB survey instrument. *Statistical Software Components*, S458353.

RAND Center for the Study of Aging. (2025). *RAND HRS Longitudinal File 2022 (V1).* Santa Monica, CA: Produced by the RAND Center for the Study of Aging with funding from the National Institute on Aging and the Social Security Administration. <https://www.rand.org/well-being/social-and-behavioral-policy/centers/aging/dataprod/hrs-data.html>
